# Non-unions treated with bone morphogenic protein 7: introducing the quantitative measurement of human serum cytokine levels as promising tool in evaluation of adjunct non-union therapy

**DOI:** 10.1186/s12950-016-0111-x

**Published:** 2016-01-22

**Authors:** Arash Moghaddam, Lisa Breier, Patrick Haubruck, Daniel Bender, Bahram Biglari, Andreas Wentzensen, Gerald Zimmermann

**Affiliations:** HTRG - Heidelberg Trauma Research Group, Trauma and Reconstructive Surgery, Center of Orthopaedics, Traumatology and Paraplegiology, Heidelberg University Hospital, Schlierbacher Landstraße 200a, D-69118 Heidelberg, Germany; Department of Orthopaedics and Traumatology, St. Marienkrankenhaus, Salzburger Str. 15, 67067 Ludwigshafen, Germany; Department for anesthesiology, Stadtklinik Frankenthal, Elsa-Brändenström Str. 1, D-67227 Frankenthal, Germany; Berufsgenossenschaftliche Unfallklinik Ludwigshafen, Department of Paraplegiology, Ludwig-Guttmann-Straße-13, D-67071 Ludwigshafen, Germany; Berufsgenossenschaftliche Unfallklinik Ludwigshafen, Trauma Center, Ludwig-Guttmann-Straße-13, D-67071 Ludwigshafen, Germany; Department for Trauma Surgery, Theresienkrankenhaus und St. Hedwigs-Klinik GmbH, Bassermannstr. 1, D-68165 Mannheim, Germany

**Keywords:** Non-union, Cytokines, BMP-7, Fracture healing, Pseudarthrosis, TGF- β, PDGF, bFGF

## Abstract

**Background:**

In this study we sought to determine if application of bone morphogenic protein 7 (BMP-7) promotes physiological bone healing of non-unions and to investigate if serum cytokine analysis may serve as a promising tool in the analysis of adjunct non-union therapy. Therefore we analyzed the influence of BMP-7 application on the serum cytokine expression patterns on patients with impaired bone healing compared to patients that showed proper bone healing.

**Methods:**

Our study involved analyzing blood samples from 208 patients with long bone fractures together with patients that subsequently developed non-unions. From this large pool, 15 patients with atrophic non-union were matched to 15 patients with atrophic non-union treated with local application of BMP-7 as well as normal bone healing. Changes in the cytokine expression patterns were monitored during the 1st, 2nd, 4th, 8th, 12th and 52nd week. The patients were followed both clinically and radiologically for the entire duration of the study. Serum cytokine expression levels of transforming growth factor beta (TGF-β), platelet-derived growth factor (PDGF) and basic fibroblast growth factor (bFGF) were analyzed and compared.

**Results:**

Serum expression of TGF-β were nearly parallel in all three groups, however serum concentrations were significantly higher in patients with proper bone healing and those treated with BMP-7 than in patients with non-unions (*p* < 0.05). bFGF serum concentrations increased initially in patients with proper bone healing and in those treated with BMP-7. Afterwards, values decreased; bFGF serum concentrations in the BMP-7 group were significantly higher than in the other groups (*p* < 0.05). PDGF serum concentration levels were nearly parallel in all groups, serum concentrations were significantly higher in patients with proper bone healing and those treated with BMP-7 than in patients with non-unions (*p* < 0.05).

**Conclusion:**

Treatment with BMP-7 in patients with former non-unions led to similar cytokine expression patterns after treatment as those found in patients with proper bone healing. Our results suggest that treatment with BMP-7 promote healing of non-unions. Furthermore, quantitative measurement of serum cytokine expression is a promising tool for evaluating the effectiveness of additional non-union therapies such as adjunct application of growth factors.

## Background

The therapy of non-unions in trauma surgery still remains a challenge [1]. Currently there are various surgical treatment options for non-unions [2], including revision surgery, medullary reaming, and dynamisation of nails. The gold standard for treating non-unions is the use of autologous bone grafts [3] harvested from the iliac crest, which have osteogenic, osteoinductive, and osteoconductive properties [4, 5]. Patients who do not respond to such therapies require further surgical interventions, such as adjunct application of bone morphogenic protein 7 (BMP-7) during revision surgery.

In the last 20 years, various attempts have been made to develop treatment plans involving growth factors for non-unions. BMP-7, a member of the transforming growth factor beta (TGF-β) - superfamily, is one of the first growth factors that was approved for clinical use. Originally applied for non-unions of the tibia, it has been used on many localizations for over 10 years without significant complications [6]. In clinical studies, BMP-7 was documented as being 80–90 % effective in healing non-unions, showing comparable or even better results as autologous bone grafting [7, 8].

Despite its potential for therapy, the success of BMP-7 on fracture healing has only been confirmed through radiological and clinical parameters in humans. A major disadvantage of these methods is that evaluation of fracture healing is only possible after at least 3 months [9], and the frequency of x-ray and CT imaging is limited due to radiation exposure. Studies on the modulating effects of BMP-7 on serum cytokine levels in animals are limited in their transferability to humans [10].

In this clinical study, we analyzed the effects of BMP-7 application during non-union therapy on human serum cytokine levels during a 1 year follow-up study. The basis for our research concept came from previous studies, in which we described several cytokine expression patterns in regular and failed bone healing [11–14]. We were able to detect significant differences in expression patterns at certain time points, which suggested that fracture healing was delayed or failed. In a further study we could show that smoking decreased the expression of TGF-β in patients after surgery [15]. From these studies we concluded that measuring serum cytokine expression is a valid instrument for evaluating bone healing [15, 16]. We chose to use this concept in order to investigate if treatment with BMP-7 may promote physiological healing of non-unions and if quantitative measurement of serum cytokine expression is a promising tool in the future for evaluating the effectiveness of additional non-union therapies such as adjunct application of growth factors.

## Methods

### Patient recruitment, clinical investigation

Two hundred and eight Patients (159 men, 49 women, aged 18 to 76 years) were enrolled in our study at BG Trauma center Ludwigshafen between 2002 and 2007. Inclusion criteria were diaphyseal fractures of long bones (humerus, radius, ulna, femur, tibia). Exclusion criteria were the existence of systemic diseases such as hypo- or hyperthyroidism, diabetes mellitus, advanced liver disease, chronic inflammatory diseases, malignancy, and extreme obesity as well as long-term use of immunosuppressive drugs, which can influence normal cytokine levels. All enrolled patients were invited to attend follow-up examinations at standardized intervals: 1, 2, 4, 8, 12 weeks and 1 year after surgery. Blood samples were taken at every follow-up examination. Patients with radiological and clinical evidence of failed fracture healing and formation of a non-union underwent revision surgery involving debridement, BMP-7 application, and re-osteosynthesis. In these cases, blood samples were taken until definitive healing of the fracture could be confirmed.

Of the 208 patients, 160 showed proper bone healing according to x-ray results, clinical stability, and pain-free weight-bearing; 48 patients suffered from non-unions, from which 13 patients received treatment with BMP-7. Furthermore, 18 patients were treated with BMP-7 in our hospital after failed primary treatment elsewhere. We included these patients in the follow-up schedule mentioned above. The study was conducted in accordance with the declaration of Helsinki and after the approval of the ethics committee of the Ruprechts-Karls-University of Heidelberg (nr. 157/2002).

### Matching

We compared three groups of patients:

1. Fracture patients developing non-unions
2. Non-union patients treated with BMP-7
3. Fracture patients with proper healing

Patients with fractures that failed to heal after primary treatment were assigned to the non-union group. After treatment with BMP-7, these Patients were assigned to the BMP-7 group. In two cases, this was not possible, so we matched two patients with two BMP-7 patients. We matched the two groups with a third group of patients with proper fracture healing. Patients were matched on the basis of five criteria: age (+/−5 years), sex, localization of fracture, type of fracture (classification by the “Association for the Study of Internal Fixation” (AO/ASIF)) and type of osteosynthesis. If more than one match was found for a non-union patient, than the patient with the most similar type of fracture was chosen. According to matching criteria three groups (*n* = 15) could be formed of the above mentioned total study patients, whereby patients that developed a non-union and subsequently were treated with BMP-7 were assigned to two groups (Table 1).

**Table 1:** Patient demographics and clinical characteristics

		Gender		Age	Type of Fracture (AO)				Lokalisation of Fracture				Fixation		
Patients	N	♀	♂		A	B	C	C1°	Femur	Tibia	Forearm	Upper arm	Nail	Plate	Ext. Fix.
All patients	45	9	36	47,37 ±11,54	30	12	15	9	12	48	3	3	27	36	3
Union group	15	3	12	47,2 ±12,00	10	4	5	3	4	16	1	1	9	12	1
Non-union group	15	3	12	47,53 ±11,48	10	4	5	3	4	16	1	1	9	12	1
BMP-7 group	15	3	12	47,53 ±11,48	10	4	5	3	4	16	1	1	9	12	1

*Abbreviations*: *BMP-7* Bone morphogenic protein 7; *Ext. Fix*. Osteosynthesis by external fixation; *AO* AO-Classification. Age is expressed as mean years ± SD

### Administration of BMP-7

Patients that showed radiological and clinical evidence of failed fracture healing and formation of a non-union during the follow-up examinations subsequently underwent revision surgery. In particular revision surgery included radical debridement and resection of avital bone tissue and furthermore re-osteosynthesis was performed, providing a better biomechanical stability. Hereafter the defect site was filled with autologous bone marrow that has an osteogenic potential, thus able of forming bone independently [17]. To maximize the effect the autologous bone marrow was enhanced by a nonrecurring local application of 3.3 mg BMP-7 [18–21].

### Sample acquisition and measuring of serum cytokines

Venous blood samples from all patients were collected during follow-ups. Samples were drawn between 8 a.m. and 11 a.m. on an empty stomach. Serum was separated, and stored at−80 °C. Serum samples were thawed and equilibrated to room temperature for at least 2 h before analysis. Measurement was set in duplicates. Serum levels of TGF-β-1, PDGF and bFGF were measured with commercially available ELISA kits (‘Quantikine®ELISA Kits’; R&D Systems, Minneapolis, MN, USA). Assays were conducted according to the manufacturer’s instructions. Levels measured 1 year after trauma defined baseline levels in the union group, whereas levels measured 1 year after failed bone healing respectively revision surgery were defined baseline in the non-union group respectively BMP-7 group.

### Statistics

Pretrial power analysis revealed a minimum size of nine patients in each group. Serum levels are expressed as mean absolute concentrations ± standard error of the mean (SEM) per time point and group. The 1 year follow-up serum levels were determined as base levels in order to facilitate analysis of our data. The Friedman-test was applied to detect significant changes over time during the entire investigative period. If significant, the Wilcoxon signed rank test for paired samples was used to recognize significant changes between the reference values and post-traumatic results within both groups. For statistical comparison of serum values at a certain time point between groups, the Wilcoxon signed rank test for paired samples was used. The confidence limit was predetermined at a level of 0.05. Statistical analysis was carried out by use of SPSS for Windows 11.0 (Norusis SPSS GmbH Inc.).

## Results

### TGF-β

**Fig. 1:**
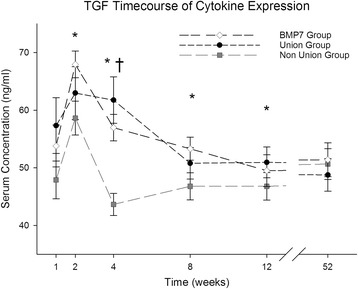
Analysis of serum TGF-β expression level. TGF-β serum level comparison over time for the entire study patients divided into groups (Union: Fracture patients with proper healing, Non-Union: Fracture patients developing non-unions, BMP-7: Non-union patients treated with BMP-7) expressed as means and SEM. Serum concentration was measured in nanogram per milliliter (ng/ml). The Wilcoxon signed rank test assessed significant differences between both groups at each particular time point (* indicates significant differences between the Non-Union and BMP-7 Group, whereas † indicates significant differences between Union and Non-Union Group *p* < 0.05)

Generally speaking, TGF-β serum concentrations increased in all groups during the first 2 weeks after surgery (BMP-7 week 1: 53.80 ± 3.63 ng/ml, week 2: 67.94 ± 2.32 ng/ml; Non-union week 1: 47.90 ± 3.29 ng/ml. week 2: 58.63 ± 2.94 ng/ml; Union week 1: 57.32 ± 4.85 ng/ml. week 2: 62.98 ± 4.59 ng/ml), decreased after the 2nd week (BMP-7 week 4: 56.98 ± 2.29 ng/ml, Non-union week 4: 43.64 ± 1.90 ng/ml, Union week 4: 61.75 ± 4.02 ng/ml), and plateaued after the 6th week (BMP-7 week 6: 54.36 ± 2.13 ng/ml, Non-union week 6: 45.90 ± 2.79 ng/ml, Union week 6: 50.72 ± 2.58 ng/ml). Serum concentrations were higher in patients with proper bone healing and those treated with BMP-7 than in patients with non-unions (Fig. 1). Patients with non-unions showed a decrease below base levels (Base level Non-union: 50.64 ± 2.67 ng/ml) after the second week. Patients who received treatment with BMP-7 and patients with proper bone healing showed less decrease in serum concentrations after the second week and concentrations remained above base levels (Base level BMP-7: 51.39 ± 2.95 ng/ml and Union: 48.75 ± 2.82 ng/ml). Differences between the union and non-union group were significant in the 4th week (*p* = 0.00) while differences between the non-union and the BMP-7 group were significant in the 2nd, 4th, 8th, and 12th week (*p* = 0.031; *p* = 0.00; *p* = 0.048; *p* = 0.026).

### PDGF

**Fig. 2:**
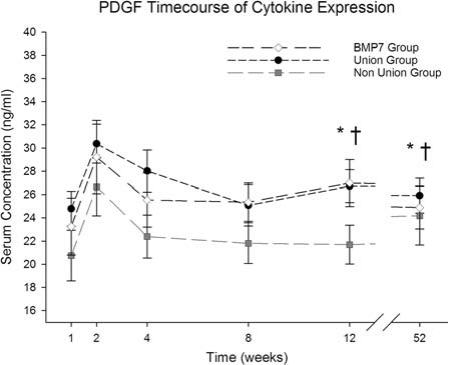
Analysis of serum PDGF expression level. PDGF serum level comparison over time for the entire study patients divided into groups (Union: Fracture patients with proper healing, Non-Union: Fracture patients developing non-unions, BMP-7: Non-union patients treated with BMP-7) expressed as means and SEM. Serum concentration was measured in nanogram per milliliter (ng/ml). The Wilcoxon signed rank test assessed significant differences between both groups at each particular time point (* indicates significant differences between the Non-Union and BMP-7 Group, whereas † indicates significant differences between Union and Non-Union Group *p* < 0.05)

PDGF serum concentration levels were nearly parallel in all three groups (Fig. 2). Values increased between the first and the second week (BMP-7 week 1: 23.25 ± 2.45 ng/ml, week 2: 29.23 ± 3.16 ng/ml; Non-union week 1: 20.75 ± 2.19 ng/ml, week 2: 26.66 ± 2.50 ng/ml; Union week 1: 24.78 ± 1.50 ng/ml, week 2: 30.38 ± 1.66 ng/ml) and decreased afterwards (BMP-7 week 8: 25.35 ± 1.65 ng/ml, Non-union week 8: 21.82 ± 1.75 ng/ml, Union week 8: 25.07 ± 1.78 ng/ml). Serum concentrations in non-union patients were lower than those in the other groups during the whole study, declining below the base value (Base level Non-union: 24.18 ± 2.51 ng/ml) after the second week without reaching this value during the following course. In the union and the BMP-7 group, values increased slightly after week 8, and differences in PDGF serum concentration between the union and the non-union groups as well as between the non-union and the BMP-7- groups were significant after week 12 (BMP-7 week 12: 26.99 ± 2.04 ng/ml, Non-union week 12: 21.70 ± 1.67 ng/ml, Union week 12: 26.73 ± 1.41 ng/ml; *p* = 0.033 and *p* = 0.041 respectively).

### bFGF

**Fig. 3:**
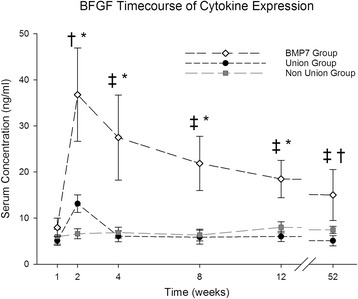
Analysis of serum BFGF expression level. BFGF serum level comparison over time for the entire study patients divided into groups (Union: Fracture patients with proper healing, Non-Union: Fracture patients developing non-unions, BMP-7: Non-union patients treated with BMP-7) expressed as means and SEM. Serum concentration was measured in nanogram per milliliter (ng/ml). The Wilcoxon signed rank test assessed significant differences between both groups at each particular time point († indicates significant differences between the Non-Union and Union Group, * indicates significant differences between the Non-Union and BMP-7 Group, whereas ‡ indicates significant differences between Union and BMP-7 Group *p* < 0.05)

bFGF serum concentrations increased in between the first and the second week in patients with proper bone healing and in those treated with BMP-7 (BMP-7 week 1: 7.96 ± 2.03 ng/ml, week 2: 36.77 ± 10.12 ng/ml; Non-union week 1: 5.94 ± 1.59 ng/ml, week 2: 6.60 ± 1.08 ng/ml; Union week 1: 5.09 ± 1.03 ng/ml, week 2: 13.12 ± 1.92 ng/ml) (Fig. 3). Afterwards, values decreased (BMP-7 week 4: 27.48 ± 9.23 ng/ml, Non-union week 4: 6.85 ± 1.18 ng/ml, Union week 4: 6.05 ± 1.19 ng/ml). Over the whole investigation period, bFGF serum concentrations in the BMP-7 group were higher than in the other groups. In non-union patients, values did not change significantly during the whole course but varied around the base level. Differences between the union and the non-union group were significant in the second week and at base levels (Base level BMP-7: 15.01 ± 5.53 ng/ml, Non-union: 7.43 ± 0.77 ng/ml, Union: 5.09 ± 1.12 ng/ml; *p* < 0.05). Differences between the BMP-7 and the non-union group were significant at week 2, 4, 8 and 12 (BMP-7 week 8: 21.86 ± 5.90 ng/ml, week 12: 18.48 ± 4.07 ng/ml; Non-union week 8: 6.32 ± 1.27 ng/ml, week 12: 7.99 ± 1.21 ng/ml; Union week 8: 5.85 ± 1.49 ng/ml, week 12: 6.01 ± 1.11 ng/ml) (*p* = 0.003; *p* = 0.011; *p* = 0.011; *p* = 0.014), differences between the BMP-7 and the union group were significant at week 4, 8, 12 and at base levels (*p* = 0.004; *p* = 0.002; *p* = 0.001; *p* = 0.026).

Serum concentrations corresponded with previous results in other studies [22–24]. Differences in serum concentrations of TGF-β, PDGF, and bFGF were significant between patients with proper bone healing and non-union patients. Results showed that the application of BMP-7 lead to expression patterns similar to those in patients with proper fracture healing. Interestingly, bFGF increased to a much higher level after week 2 in the BMP-7 group compared to other groups. Generally speaking, TGFβ, PDGF and bFGF concentrations peaked after week 2 and decreased afterwards, and were lower in patients with non-unions.

## Discussion

In this prospective, controlled clinical study, we analyzed the serum concentrations of TGF-β, PDGF and bFGF during a 1 year follow-up in non-union patients treated with BMP-7 and compared them to the cytokine expression pattern of patients with failed as well as with physiological bone healing to determine whether the local application of BMP-7 modulates the microenvironment in the fracture gap and thereby influences secondary bone regeneration.

### TGF-ß

Recent studies in fracture healing showed that successful fracture healing is a complex process involving the growth and differentiation of mesenchymal stem cells, regulation of cytokines (inflammatory and osteogenic) and the resorption of extracellular matrix [25]. The golden standard in non-union therapy is the transplantation of autologous bone graft [26–29], more recently this therapy was combined with the application of BMP-7 and studies show a better outcome after non-union treatment if BMP-7 was applied [18, 30, 31]. The underlying mechanism of the clinical success of BMP-7 application remains uncertain. In the current study we sought to determine the influence of local BMP-7 application on successful bone healing utilizing quantitative serum measurement of osteogenic cytokine expression pattern. Transforming growth factor beta is initially secreted from thrombocytes in areas of hematoma after injury, but later from chondrocytes and osteoblasts during fracture healing. It stimulates the proliferation of different cells including osteoblasts, chrondroblasts, and chondrocytes [22, 23]. Regarding the serum concentration of TGF-ß our results showed that patients in the BMP-7 group had lower levels after the first week compared to the group with physiological bone healing suggesting that osseous biology was still impaired at this time. However local application of BMP-7 lead to an increase in TGF-β after the second week that lasted longer than in every other group. It has been shown that the concentrations of bone morphogenic proteins were decreased in patients suffering from non-union, thereby indicating that a down-regulation in expression of osteogenic BMPs might be responsible for failed fracture healing [32, 33]. In our study patients with failed fracture healing were treated with adjunct application of BMP-7 serving as a substitution of known lower BMP concentrations in patients with failed fracture healing, thereby modulating the microenviroment in the fracture gap and increasing local concentration of BMP-7. BMPs and TGF-ß are both members of TGF-ß protein superfamily and promote bone formation. The most important effect of TGF-ß is the stimulation of the synthesis of components of the extracellular matrix (e.g. collagen I and III). This has been shown to be pivotal in the early stages of bone healing as scaffolding for later bone mineralization [34]. BMPs facilitate osteogenic differentiation due to various pathways in order to achieve successful bone healing [34]. Peak concentrations in the BMP-7 group were measured after week 4, suggesting that BMP-7 was able to stimulate osteoblast biology and lead to higher expression of TGF-β. BMP-7 appears to have its most significant impact between week 2 and 4 [35]. A steeper decrease in the non-union group between week 2 and 4 compared to other groups could be attributed to a decrease in hard callus formation, which plays a crucial role in non-union formation. In our previous publications, we attributed this decrease to hard callus formation between week 2 and 4 and proposed this phase to be crucial in nonunion formation. It is known that TGF-β plays an important role in the formation of cartilage-like tissue, whereas a decrease in the expression of TGF-β accounts for an impaired potential in the formation of cartilage-like tissue. Hereby indicating that impaired expression of TGF-β, as seen in decreased plasma levels, may provide a predisposing factor of an dysfunctional cartilage and osseous regeneration [24].

In conclusion it is known that local and systemic concentrations of different osteogenic cytokines increase during physiological fracture healing, in particular sufficient serum concentrations of TGF-ß have been shown to be pivotal to fracture healing [36]. It has been reported that TGF-ß and BMPs are capable of inducing each others expressions [33, 34], however patients with failed bone healing are known to have impaired local BMP concentration, furthermore the current study showed that the concentration of TGF-ß in concerned patients is decreased during the initial 12 months; indicating that BMP-7 and TGF-ß have to be present in fracture healing in a sufficient concentration [37]. Our results show that local application of BMP-7 serves as a substitution of impaired BMP-7 levels in non-union patients and thereby modulating the microenviroment in the fracture gap and promoting bone regeneration. The results of our current study accentuate the findings of previous studies indicating that lower circulating levels of TGF-ß could be utilized as a predictor of delayed bone healing and non-union [11].

### PDGF

Platelet derived growth factor was first discovered in thrombocytes, but was later found in granulation tissue, hypertrophic chondrocytes, osteoblasts, and osteoclasts during bone healing. PDGF has got many effects on fracture healing: it releases mesenchymal stem cells from blood vessels and is a potent mitogen for mesenchymal stem cells (MSC) [38, 39]. It also promotes angiogenesis by increasing the secretion of vascular endothelial growth factor (VEGF) [12, 40]. PDGF deficiency may lead to impaired bone healing as shown in diabetic animals [41]. After PDGF application in osteoporotic animals, improvement was seen in bone healing. Furthermore, PDGF application improved bone healing in diabetic patients [40], more recently published data indicates promising results in the treatment of osteochondral defects of the talus, hereby application of PDGF provides a benefit in bone healing [42].

Our findings on PDGF concentration levels in the union and the non-union group are supported by previous studies [12]. Union patients had higher levels at all time points than non-union patients. Levels increased in both groups after surgery, peaked after the fourth week, and decreased thereafter. Similar to TGF-β, levels decreased steeper in non-union-patients as in other groups. We explained this by hard callus formation as explained above. This would be supported by previous research showing that PDGF application led to an increase in callus density and volume in animal models [43]. Patients treated with BMP-7 had higher levels of PDGF than non-union patients; which is possibly due to increased PDGF secretion from BMP-7 stimulation. There was an increase in the union and the BMP-7 group between week 8 and 12, which was not detected in non-union patients. This phase of bone healing is associated with remodeling, which could be influenced by PDGF. Non-unions may not have reached the remodeling phase, resulting in reduced cytokine levels. In conclusion local application of BMP-7 increases PDGF level to a physiological extent, thereby BMP-7 substitution enhances mitogenesis of MSCs in the initial phase of bone healing, concluding in a possibly higher concentration of MSC in the fracture gap leading to favorable fracture healing. Furthermore BMP-7 application leads to an increased angiogenesis in the initial phase of fracture healing by an enhanced expression of PDGF and bFGF. The results of our study indicate that application of BMP-7 improves bone healing by enhanced angiogenesis, mitogenesis and osteogenesis.

### bFGF

Angiogenic growth factors are mainly expressed during the early phases of fracture healing whereas osteogenic growth factors are continuously expressed during bone healing and remodeling [44]. Basic fibroblast growth factor plays an important role in the angiogenic response after fracture healing [45], as well as in general bone healing [46, 47]. Apparently due to complex processes: bFGF abrogated the bone stimulating effects of BMP-7 in vitro [48, 49], and reversibly inhibited osteogenesis in human adipose-tissue derived stromal cells [50]. BFGF retains MSCs in a more “immature” state and MSC lines responding to bFGF stimulation secrete factors which provided an environment for the first stage of fracture repair [51]. Consequently, one putative function of bFGF could be the retention of mesenchymal stem cells during the first phases of fracture healing before ossification takes place. Up to date multiple combination of osteogenic and angiogenic growth factors has been used and studied in combination, however the combination of BMPs and bFGF have been one of the most popular growth factors used [52, 53]. Regarding the effect of the combined application of BMP and BFGF contradictory conclusions have been found, in particular studies have shown stimulatory and inhibitory effects [44]. In the present study, bFGF levels did not alter in non-unions while significant increases in the union groups were observed after the second week. This suggests that bFGF plays a crucial role during the first stages of fracture repair possibly as an angiogenic growth factor. BMP-7 application led to an enormous increase in bFGF expression and concentrations remained significantly higher even 1 year after surgery. In particular BMP-7 application lead to an enormous increase in the initial phase of bone healing, indicating that BMP-7 application leads to an enhanced angiogenesis in the early stages of bone healing by increasing local bFGF concentrations, furthermore bFGF levels were increased during the whole period of time, thereby promoting bone healing by a supplementary osteogenic effect during initial bone healing and later remodeling. In conclusion the results of our study accentuate a favorable effect of increased local bFGF and BMP-7 concentrations on bone healing; either due to direct stimulation of angiogenesis and osteogenesis due to BMP-7 application evaluated by increased bFGF serum level or due to an initial stimulation of bFGF expression and subsequently a co-stimulatory effect of increased local BMP-7 and bFGF concentrations. These findings stress the significance of a molecular link between BMP and bFGF signal pathways [54].

### Limitations

Non-unions are a severe but an infrequent complication resulting in clinical studies concerning non-union therapy being complex and it explains the small size of our patient collective. Despite a large patient collective just a small number of patients could be included in the study due to our strict inclusion and exclusion criteria. In the context of current literature and our recent studies [14, 55], our patient collective size is still sufficient. Another limitation of this study may arise from the different age of samples, samples were collected throughout the whole study period, however measurements were performed cumulated for all samples, which could influence the cytokine concentration. However, since samples of all groups were concerned equally we believe that the sample age does not interfering with the results of our study. The strengths of this study are its prospective approach, the clear inclusion and exclusion criteria, and the matching of patients. This allowed us to eliminate factors that could bias the evaluation of these patient groups.

## Conclusions

In this prospective, controlled study, we analyzed the serum concentrations of TGF-β, PDGF and bFGF during a 1 year follow-up in non-union patients treated with BMP-7. We compared them to the cytokine expression pattern of patients with failed and with proper bone healing to determine if the local application of BMP-7 modulates the microenvironment in the fracture gap and thereby influences secondary bone regeneration. The results of our study show that local application of BMP-7 lead to a cytokine expression pattern of bone healing associated cytokines (TGFβ, PDGF and bFGF) that resembled the expression patterns of patients with proper bone healing and differed significantly from those in patients developing non-unions. Concluding in the current study we could show that patients that develop non-unions have an abnormal cytokine expression pattern of osteogenic cytokines, in particular serum cytokine levels were decreased beginning at the start of fracture healing. Local application of BMP-7 elevated serum cytokine level to a physiological extent and thereby facilitated bone regeneration by enhanced angiogenesis, mitogenesis and osteogenesis. We believe our study adds important knowledge to the literature in the field by providing a possible mechanism of how adjunct application of BMP-7 promotes bone healing gathered from clinical data.

Furthermore this present study showed that quantitative measurement of serum cytokine expression is a valid instrument in evaluating the biological impact of BMP-7 and thus the value of possible non-union therapies. Further studies using this protocol to determine serum cytokine expression levels will help elucidate the effectiveness of other non-union therapies, and compare these therapies in order to make a biology-based decision for the treatment of non-unions.

### Ethics, consent and permission

Patients were included in our study after giving a full written declaration of consent, including the use of the gathered data, as well as the consent to publish the data acquired. The study was conducted in accordance with the declaration of Helsinki and after the approval of the ethics committee of the Ruprechts-Karls-University of Heidelberg (nr. 157/2002).

## Abbreviations

BMP-7: Bone morphogenic protein 7
BMP: Bone morphogenic protein
TGF-β: Transforming growth factor beta
PDGF: Platelet-derived growth factor
BFGF: Basic fibroblast growth factor
AO/ASIF: Association for the study of internal fixation
SEM: Standard error of the mean
MSC: Mesenchymal stem cell
VEGF: Vascular endothelial growth factor
